# Sexual Interest and Arousal in Males Who Are Civilly Committed: Exploring Overlap and Distinctions

**DOI:** 10.1007/s10508-026-03547-6

**Published:** 2026-09-19

**Authors:** Melinda R. Ward, Ashley E. Thompson, Timothy G. Thornton

**Affiliations:** 1 Direct Care and Treatment, Minnesota Sex Offender Program, Vadnais Heights, MN USA; 2 Department of Psychology, University of Minnesota Duluth, 347B BohH 1207 Ordean Court, Duluth, MN 55812 USA

**Keywords:** Sexual arousal, Sexual interest, Deviance differential, Adult sexual abusers, Penile plethysmography, DSM-5

## Abstract

Accurate assessment of sexual deviance is critical for treatment planning and risk management among individuals who have committed sexual offenses. Penile plethysmography (PPG) is often regarded as the most specific measure of male physiological sexual arousal, though concerns about invasiveness and standardization have prompted interest in less intrusive alternatives. Viewing time (VT) paradigms index sexual interest through differences in cognitive processing latency when evaluating stimuli, yet few studies have directly compared VT outcomes to PPG findings. This study examined the relationship between PPG-derived deviance differentials (DD) and VT mean rank scores in 146 civilly committed adult males. Participants were classified into child arousal, undifferentiated arousal, or adult arousal groups based on PPG DD scores, and VT category-level mean rank scores were compared across developmental and gendered stimulus categories. Significant group differences emerged for female stimuli only. Individuals in the child arousal group exhibited longer viewing times for images of small child and pre-juvenile female images, whereas those in the adult arousal group exhibited longer viewing times for adult women. These findings provide converging evidence that VT aligns with PPG-derived classifications, supporting a multi-method assessment framework.

## Introduction

Understanding sexual deviance among individuals who have committed sexual offenses is critical for effective risk assessment, treatment planning, and prevention of recidivism. The assessments most frequently used to understand, explore or identify sexual deviance are physiological measures of arousal, particularly penile plethysmography (PPG) (Hanson & Bussière, 1998; McPhail et al., 2019; Schmidt et al., 2017; Wilson & Miner, 2016). However, there is growing interest in the integration of less invasive cognitive measures, such as viewing time (VT), to capture latency-based indicators of sexual interest (McGrath et al., 2012; Mokros et al., 2013; Thornton & Laws, 2009). Thus, the current study sought to explore the relationship between physiological sexual arousal and cognitive indicators of sexual interest by examining how PPG-derived deviance differentials correspond with relative VT across stimulus categories.

### Definition of Terms

We recognize that terms are not used consistently in the literature. To that end, the following section defines key terms related to the assessment and classification of sexual arousal, interest, and deviance.

### Deviance Differential (DD)

The difference in an individual’s arousal response to sexually deviant stimuli versus normative stimuli.

### DD Groups

#### Adult Arousal

Individuals who exhibit greater physiological arousal to adult stimuli compared to prepubescent/pubescent stimuli.

#### Child Arousal

Individuals who exhibit greater physiological arousal to prepubescent/pubescent stimuli compared to adult stimuli.

#### Undifferentiated Arousal

Individuals who show relatively similar arousal patterns to both prepubescent/pubescent and adult stimuli without a preference.

### Penile Plethysmography (PPG)

A genital measure of sexual arousal that records changes in penile circumference or volume in response to sexual stimuli.

### Sexual Arousal

A physiological response of penile engorgement as measured by PPG.

### Sexual Deviance

A sexual interest in or arousal by “activities, situations, people, or objects that are illegal, inappropriate, or highly unusual” (Fernandez et al., 2014, p. 91). These include, but are not limited to, those directed toward individuals unable to give legal consent (e.g., children).

### Sexual Interest

The cognitive and emotional attention directed toward specific sexual stimuli, often assessed via viewing time measures.

### Viewing Time (VT)

A cognitively based, indirect measure of sexual interest (Pedneault et al., 2021). The stimulus response time or on-task latency (OTL; i.e., the length of time a participant spends viewing, evaluating, and rating sexually salient images) is unobtrusively recorded during the assessment. Although other terms, such as visual reaction time (VRT^™^) and visual response time, have been used when describing this type of assessment, this paper will use VT to refer specifically to viewing time paradigms and corresponding OTL measures.

### The Measurement of Sexual Deviance

Accurate assessment of sexual deviance is vital to the evaluation and treatment of individuals who have committed, or are at risk of committing, sexual offenses against children (Abracen & Looman, 2015; McPhail et al., 2019; Thornton et al., 2018). Tailoring interventions requires a thorough assessment of sexuality, particularly when addressing risk of re-offense. In forensic settings, sexuality is typically assessed via measures of sexual interest and sexual arousal. Researchers and practitioners have used the VT paradigm as an indirect index of sexual interest in which individuals view images representing varying sex and sexual maturity categories while completing a task (e.g., rating images). Software tracks how long participants view each image, with longer viewing times inferred to reflect greater sexual interest (Schmidt et al., 2017). Research examining the processes underlying the latency patterns observed in VT assessments has proposed that these effects may be influenced more by task demands than by the stimulus itself (Imhoff et al., 2012). Although initially designed to investigate sexual orientation (e.g., Israel & Strassberg, 2009; Rönspies et al., 2015), VT has since been applied to sexual preferences based on sexual maturity and is a common measure of sexual interest in children in forensic settings (McGrath et al., 2012). Research demonstrates that VT tasks reliably distinguish men who have committed sexual offenses against children from those who have not (Schmidt et al., 2017).

Measuring sexual arousal provides another method of assessing sexual deviance. Various psychophysiological measures have been studied (e.g., thermistor, heart rate, pupillary response), but the PPG, which measures genital arousal, is recognized as the “most specific measure of sexual response in men” (Chivers et al., 2010, p. 8; Kalmus & Beech, 2005; Plaud, 2019; Thornton et al., 2018; Zuckerman, 1971). The PPG, which quantifies erectile responses to sexual stimuli, is used internationally for diagnosis, treatment planning, and risk management (Wilson & Miner, 2016). Phallometric stimuli may include images, audio descriptions, video, or computer-generated images (Kalmus & Beech, 2005; Renaud et al., 2013). Meta-analyses underscore the utility of the PPG in predicting recidivism. Hanson and Bussière (1998), for instance, found arousal to children to be the strongest single predictor of recidivism, while McPhail et al. (2019) confirmed that phallometric assessments distinguish individuals who have committed sexually abusive behaviors from controls and significantly predict recidivism.

Despite its clinical value, the PPG has been criticized for invasiveness (Bernstein, 2016; Wilson & Miner, 2016), potential discomfort or embarrassment (Merdian & Jones, 2011), and lack of standardization in stimuli, equipment, and interpretation (Carvalho et al., 2020; Mackaronis et al., 2016; Marshall, 2014; Murphy et al., 2015, 2021; Thornton et al., 2018; Wilson & Miner, 2016; Yates, 2021). Timing and suitability also limit its use; age, mental and physical health may preclude participation, and not all programs have access to necessary resources or trained personnel. The Association for the Treatment and Prevention of Sexual Abuse (ATSA) (2025) guidelines recommend considering client willingness and motivation, timing of evaluation, and ensuring that clients understand the purpose and impact of the assessment. Importantly, clinical diagnoses, decisions regarding supervision or placement, and treatment outcomes should never be based solely on PPG results; instead, best practice involves integrating multiple sources when assessing sexual deviancy, informing treatment planning, and estimating recidivism risk (ATSA, 2025; Carvalho et al., 2020; Marshall & Fernandez, 2000; Plaud, 2019).

### Deviance Index/Differential

An area that lacks standardization is the analysis, classification, and utilization of phallometric data. A deviance index or deviance differential (DD), which reflects the difference in arousal between sexually deviant and normative stimuli, is commonly used by practitioners and researchers across legal, clinical, and forensic settings. However, the computation and interpretation of the DD varies across studies. Some calculate it using raw scores or z-scores, either by subtracting or dividing responses to different categories (Barbaree & Marshall, 1988; Harris et al., 2003; Mackaronis et al., 2016; Nunes et al., 2002; Rice et al., 1991; Schober, 2008). While some researchers use cutoff scores to dichotomize participants into pedophilic or non-pedophilic groups (Blanchard et al., 2001; Gretton et al., 2001), others treat the DD as a continuous variable (Castro-Calvo et al., 2023). For this study, the DD provided by the Monarch 21 PPG (Behavioral Technology, Inc., Salt Lake City, UT) software was used to classify participants into three groups: (1) child arousal, (2) undifferentiated arousal, or (3) adult arousal. The undifferentiated arousal group, typically not considered in research, was included in the analyses.

### Relationship Between Measures of Sexual Arousal and Interest

Although VT has been proposed as a complementary, less invasive alternative to the PPG, relatively few studies have examined its concordance with PPG or whether these methods index overlapping aspects of sexual deviance. For instance, Gray and Plaud (2005) found consistent results across PPG and VT among individuals diagnosed with pedophilia, and Harris et al. (1996) reported that VT differentiated child molesters from controls using a deviance index. The average mean PPG responses to child stimuli also exceeded adult responses among individuals who offended against children. Among adolescents, Mackaronis et al. (2016) observed significant, albeit weak, positive correlations between raw PPG and VT scores, while Letourneau (2002) found positive correlations for both child and rape-related stimuli. In contrast, Babchishin et al. (2014), using calculated differential scores for PPG and VT, found a negative, nonsignificant correlation, raising questions about the extent to which these modalities capture overlapping versus distinct components of sexual deviance.

Although some research has shown associations between PPG and VT, no studies have compared sexual interest across the three DD groups. Incentive motivation theory (IMT; Toates, 2009) provides a framework for integrating PPG and VT, positing that sexual stimuli acquire salience through conditioning and reinforcement, forming motivational hierarchies that operate at automatic and controlled levels. In this view, PPG captures a physiological endpoint, whereas VT reflects latency-based cognitive processing during structured stimulus evaluation, such that stimuli of greater sexual relevance/interest require more extensive processing (Imhoff et al., 2012). Accordingly, VT should diverge across DD groups in ways that mirror underlying patterns of sexual salience.

### The Current Study

The object of the current study was to explore the relationship between physiological sexual arousal and indices of relative sexual interest across stimulus categories, as assessed using PPG and a rank-scored VT measure, in adult male clients who have been civilly committed (that is, individuals court-ordered to a secure treatment facility following incarceration due to a determination that they pose an ongoing risk of sexual harm, and held for an indeterminate duration). While DDs have been used to classify individuals into clinically meaningful groups (e.g., Blanchard et al., 2001; Gretton et al., 2001), research is needed to explore the characteristics that differentiate these groups. Accordingly, this study was designed to examine whether distinct patterns of sexual interest, as inferred from relative differences in stimulus–response latencies, emerge across three groups of individuals categorized by their DD: (1) child arousal, (2) undifferentiated, or (3) adult arousal. The inclusion of VT data offers a novel lens through which to assess the convergent validity of the DD classifications. Although no specific hypothesis was generated for the undifferentiated group, given the lack of prior research on this group, they were included in the analyses to provide some preliminary insight into their VT tendencies.

Thus, using IMT as a theoretical framework, the following hypotheses were generated:

H1: Individuals classified in the child arousal group were expected to exhibit longer viewing times to pre-juvenile and small child stimuli relative to those in the adult arousal group, consistent with a deviant sexual interest profile.

H2: Individuals classified in the adult arousal group were expected to exhibit longer viewing times to adult stimuli than individuals in the child arousal group, consistent with a normative sexual interest profile.

## Method

### Participants

The initial sample comprised 253 assessments for 237 individuals (all assigned male at birth) who were civilly committed in the Minnesota Sex Offender Program as a “Sexually Dangerous Person” (i.e., someone who is likely to engage in acts of harmful sexual conduct) and/or “Sexual Psychopathic Personality” (an individual who lacks the power to control their sexual impulses; MN Statutes 253D.02). Commitment to this treatment facility followed incarceration and clinical evaluations indicating that the individual had a documented history of harmful sexual conduct and posed an ongoing threat of future sexual harm. To be included in the sample, clients had to have a PPG and VT assessment completed within 30 days of one another and a calculated DD.

An embedded attention task was used to assess engagement during the PPG assessment. Clients were instructed to press a button when a star was periodically displayed during the visual stimuli and when a tone was presented during the audio portion. An attention percentage was calculated as the number of appropriately timed button presses divided by the total number of stars/tones presented, with values truncated to the nearest integer. Additionally, the assessment reports and technician notes for the nine clients whose PPG attention percentage was below 80% or unavailable due to technical difficulties were reviewed by the research team. It was decided that four participants would be retained as there were no notes regarding client movement, inattention, or looking away from the images and assessment psychologists interpreted their assessments results as clinically valid. For clients who completed the data collection procedure on multiple occasions, only data from their first administration during the study period were retained to help mitigate the influence of treatment. With these criteria in mind, a total of 107 assessments were removed, resulting in a final sample of 146 participants.

The age of the sample ranged from 27 to 74 years at the time of assessment (*M* = 47.59, *SD* = 10.87), and 69.18% identified as heterosexual. The majority identified as White (73.29%) and had never been married (65.07%). On average, participants had been committed for a total of 12.62 years at the time of assessment (*SD* = 5.75), and the majority reported having at least one victim younger than 11 (74.66%). For the demographic variables, the participants in the undifferentiated arousal DD group tended to resemble those in the adult arousal group more than the child arousal group. Table 1 presents the demographic information of the sample.

**Table 1:** Demographic information by deviant differential groups

	Child arousal	Undifferentiated arousal	Adult arousal	Total sample
	*N*(%)/*M*(*SD*)	N(%)/*M*(*SD*)	N(%)/*M*(*SD*)	N(%)/*M*(*SD*)
Number of participants	62	39	45	146
*Race*				
White	49 (79.03%)	27 (69.23%)	31 (68.89%)	107 (73.29%)
Non-white/other	13 (20.97%)	12 (30.77%)	14 (31.11%)	39 (26.71%)
Age at PPG (in years)	50.71 (10.82)	45.61 (10.87)	45.01 (10.05)	47.59 (10.87)
*Marital status*				
Never married	34 (54.84%)	28 (71.79%)	33 (73.33%)	95 (65.07%)
Previously/currently Married	28 (45.16%)	11 (28.21%)	12 (26.67%)	51 (34.93%)
*Sexual preference*				
Heterosexual	40 (64.52%)	32 (82.05%)	29 (64.44%)	101 (69.18%)
Bisexual	18 (29.03%)	7 (17.95%)	13 (28.89%)	38 (26.03%)
Homosexual	3 (4.84%)	0 (0.00%)	2 (4.44%)	5 (3.42%)
Unsure	1 (1.61%)	0 (0.00%)	1 (2.22%)	2 (1.37%)
Time committed (in years)	12.88 (5.72)	12.48 (5.61)	12.40 (6.03)	12.62 (5.75)
*Pedophilic disorder diagnosis*				
Yes	44 (70.97%)	17 (43.59%)	10 (22.22%)	71 (48.63%)
No	18 (29.03%)	22 (56.41%)	35 (77.78%)	75 (51.37%)
*Victim age(s)*				
Infant, prepubescent and/or pubescent^*^	56 (90.32%)	26 (66.67%)	27 (60.00%)	109 (74.66%)
Teen or adult only	6 (9.68%)	13 (33.33%)	18 (40.00%)	37 (25.34%)
*Gender of victim(s)*				
Both	31 (50.00%)	14 (35.90%)	21 (46.67%)	66 (45.21%)
Female	29 (46.77%)	23 (58.97%)	22 (48.89%)	74 (50.68%)
Male	2 (3.23%)	2 (5.13%)	2 (4.44%)	6 (4.11%)
*Total number of PPGs*				
1	33 (53.23%)	23 (58.97%)	24 (53.33%)	80 (54.79%)
2	13 (20.97%)	3 (7.69%)	12 (26.67%)	28 (19.18%)
3	12 (19.35%)	9 (23.08%)	5 (11.11%)	26 (17.81%)
4 +	4 (6.45%)	4 (10.26%)	4 (8.89%)	12 (8.22%)

^*^Reported at least one victim, grammar pubescent. Teen and/or adult victims could be included as well

### Procedure

After receiving approval from the institutional review board for the Minnesota Department of Human Services, clients who had completed the assessments of sexuality were eligible for the current study. The assessments were administered by trained research analysts as part of routine program treatment; therefore, no payment was provided. The typical data collection procedure required clients to review and sign a consent form in which they agreed to voluntarily participate in the assessments of sexuality, fill out a pre-test questionnaire, complete the Affinity assessment, the PPG assessment, and a post-test questionnaire. While most participants finished VT and PPG testing on the same day, nine (6.16%) completed the assessments on different days. All data for the study were collected between August 1, 2017, and August 31, 2023.

### Measures

#### Penile Plethysmography

Physiological arousal was assessed using the Monarch 21 PPG system, with the standard set of audio and visual stimuli. The Monarch 21 system operates via an Indium-Gallium circumferential strain gauge that is placed around the client’s penis, which detects minute changes in penile tumescence during stimulus presentation. These physiological responses are recorded continuously throughout the assessment and analyzed to determine patterns of sexual arousal across stimulus categories (see Castro-Calvo et al., 2023 for detailed testing procedures).

During the assessment, clients first viewed a neutral segment to measure baseline arousal. Then, 21 segments were presented, followed by a minimum 25-s period for detumescence (during which time the client did not see or hear anything). For each segment, clients viewed an image, followed by a 90-s audio narrative related to the sex and sexual maturity of the image. The narratives were voiced by an adult male and depicted persuasive (sexually nonviolent) or coercive (sexually violent) scenarios. Lastly, clients were shown four additional images for 10-s each of individuals that matched the segment's sexual maturity and sex. The stimulus sets of interest for the current study included prepubescent, pubescent, and adult categories.

For scoring purposes, DDs were calculated to capture relative patterns of physiological arousal across stimulus categories. Specifically, DD values were computed as the difference between mean standardized responses (*z*-scores) to adult stimuli and mean standardized responses to child stimuli, where each mean incorporated both maximum response and area under the curve indices. Thus, DD scores reflect relative arousal (adult minus child), with negative values indicating greater arousal to child stimuli and positive values indicating greater arousal to adult stimuli.

To ensure sufficient physiological responsiveness, at least one significant penile response (i.e., an increase of 0.47 cm from baseline) during the 16 core stimulus sets was required to compute a DD. Each core stimulus set included persuasive and coercive segments for both sexes across prepubescent, pubescent, teen, and adult stimuli. Although responses from the teen segments were not included in the DD calculation, a significant penile response to a teen segment permitted computation of a DD.

DD values were then used to classify individuals into groups reflecting child arousal (DD ≤ − 0.30), adult arousal (DD ≥ 0.30), or undifferentiated arousal (DD − 0.29 to 0.29). These cut points were selected based on their common use in applied forensic contexts. Specifically, similar classification thresholds are routinely implemented across numerous civil commitment programs in the United States to inform risk assessment and treatment planning decisions. Accordingly, their use in the present study enhances ecological validity and ensures alignment with prevailing clinical practice.

#### Viewing Time

The Affinity 2.5 (Pacific Behavioural Assessment, Victoria, BC) is a computerized assessment designed to indirectly measure sexual interest while participants view and rate images of clothed individuals. The software presented 80 standardized images depicting individuals at four developmental stages (i.e., small children, pre-juveniles, juveniles, adults), spanning both male and female images (10 in each category). To begin, participants clicked a button, after which a red circle appeared to orient visual attention. A visual analog scale also appeared for participants to rate how sexually attractive or unattractive they found each image. Following a brief pause, the circle disappeared, and an image was displayed. The program then began to unobtrusively record VT to calculate the OTL (i.e., the time the participant takes to make each rating). OTL scores were computed using the program’s ipsative mean-rank procedure. For each participant, the raw stimulus latencies for all images were ranked, tied ranks were averaged, and ranks were summed for each category and divided by the number of images (10) to create a category-level mean rank OTL score. Scores therefore reflect relative within-person processing latency rather than absolute viewing time. The OTLs for juvenile images were not included in the analyses to be consistent with the DD calculation.

#### Demographic Information

Race, date of birth (used to calculate age at the time of assessment), marital status, commitment date, and pedophilic disorder diagnosis were all extracted from the case management system that stores clients’ data. The total number of PPG assessments was obtained from assessment records. Clients self-reported their sexual preference on the pre-test questionnaire by circling one of the following options: exclusively heterosexual, bisexual, exclusively homosexual, or unsure. Additionally, they reported the sex and sexual maturity of their victim(s). Sexual maturity was categorized as: 0 to 2 years old—infant, 3 to 5 years old—prepubescent, 6 to 10 years old—pubescent, 11 to 17 years old—teen, and 18 +—adult.

## Results

Prior to conducting the analyses, all variables were screened for univariate outliers. Outliers were assessed by computing z-scores for each variable, with values exceeding ± 3.00 identified as potential outliers (Tabachnick & Fidell, 2019). Examination of the six values that met the outlier criteria indicated that most were continuous with the overall data distribution and did not reflect extreme deviations. As the identified outliers did not substantively influence data integrity or distributional shape, they were retained in all subsequent analyses (Aguinis et al., 2013).

Additionally, skewness was assessed by computing skew z-scores and applying a cutoff of ± 2.58. Five mean ranked OTL variables exceeded this threshold and demonstrated significant skew: Adult Female (negatively skewed), Adult Male, Pre-Juvenile Male, Small Child Female, and Small Child Male. After reverse scoring the Adult Female variable, skew for each measure was corrected using square root transformations (Adult Female, Adult Male, and Small Child Female scores) or log10 transformations (Pre-Juvenile Male and Small Child Male scores). The transformed variables were used as dependent variables in all parametric analyses, whereas raw descriptive statistics are reported for interpretability.

After completing data screening and cleaning, descriptive statistics for category-level OTL mean rank scores were calculated for each DD group. In general, those in the adult arousal group demonstrated higher relative OTL scores for adult stimuli, whereas those in the child arousal group demonstrated higher relative OTL scores for child stimuli, with the undifferentiated group exhibiting intermediate values. For detailed descriptives, see Table 2. To provide additional evidence of convergent validity, exploratory analyses were conducted examining correlations between absolute and relative indices of sexual interest and arousal (to review, visit our OSF page: https://osf.io/764xj/overview?view_only=00d56ac1acf54a8b8f781ff0abb5bc86).

**Table 2:** Means and standard deviations for Affinity On-Task Latency by Deviance Differential group

	Child arousal	Undifferentiated arousal	Adult arousal
	*N*(%)/*M*(*SD*)	*N*(%)/*M*(*SD*)	*N*(%)/*M*(*SD*)
Number of participants	62 (42.47%)	39 (26.71%)	45 (30.82%)
*OTL—mean rank scores*			
Adult female	53.21 (11.05)	58.71 (9.43)	**61.20 (10.53)**
Adult male	33.43 (12.24)	33.00 (9.73)	**38.92 (13.46)**
Pre-juvenile female	**44.07 (10.61)**	41.20 (9.91)	38.37 (10.35)
Pre-juvenile male	**33.55 (8.18)**	29.79 (6.93)	30.79 (8.61)
Small child female	**40.41 (11.61)**	36.99 (9.38)	31.17 (7.39)
Small child male	**26.49 (8.31)**	25.26 (7.51)	24.88 (6.76)

Highest OTL—mean rank scores in each affinity category are bolded

To examine the differences in sexual interest inferred by mean rank OTLs between DD groups, six one-way analyses of variance (ANOVAs) were conducted. To mitigate the risk of an inflated Type I error rate due to multiple omnibus tests, a Bonferroni correction was applied to adjust the significance threshold. This resulted in an adjusted *p* value of 0.008 (0.05/6), which was used to evaluate the statistical significance of all reported effects. Additionally, when the assumption of homogeneity of variance was violated, Welch’s test was employed as a robust alternative to the standard ANOVA, as it does not assume equal variances across groups. Finally, all significant effects were followed up with one planned contrast comparing the adult arousal DD group to the child arousal DD group (with the undifferentiated arousal DD group retained in the model).

For the results of the ANOVAs, a significant effect of DD group emerged for the adult female (*F*[2, 143] = 9.14, *p* < 0.001, *η*_*p*_^*2*^ = 0.11) and small child female Affinity stimuli (*F*[2, 90.92] = 12.42, *p* < 0.001, *η*_*p*_^*2*^ = 0.14). The follow-up planned contrasts revealed a significant difference between the adult arousal DD group and the child arousal DD group for both adult female (*t*[143] = 4.19, *p* < 0.001, *d* = 0.82) and small child female (*t*[104.98] = 4.85, *p* < 0.001,* d* = 0.93) stimulus scores. Although the omnibus test was not significant for the pre-juvenile female stimuli, planned contrasts were conducted as the difference in means suggested a possible significant difference, which was supported (*t*[143] = 2.81, *p* = 0.006, *d* = 0.55). Consistent with our hypotheses, individuals in the child arousal DD group demonstrated higher OTL mean rank VT scores for images of small child and pre-juvenile females, whereas those in the adult arousal DD group demonstrated higher mean rank scores for images of adult females. For all six omnibus test results, please see Table 3.

**Table 3:** ANOVA results for affinity On-Task Latency

	*df*	*F*	*p*	*ηp2*
OTL—Mean rank scores				
Adult female	2, 143	9.14	< 0.001*	0.11
Adult male	2, 143	3.21	0.04	0.04
Pre-juvenile female	2, 143	3.98	0.02	0.05
Pre-juvenile male	2, 143	3.42	0.04	0.05
Small child female	2, 90.92	12.42	< 0.001*	0.14
Small child male	2, 143	0.42	0.66	0.01

^*^^adjusted *p* < 0.008^

## Discussion

The current study examined whether sexual interest, inferred from a VT measure, varied according to physiological indicators of sexual arousal assessed via PPG. The results indicated that individuals classified into the child or adult arousal DD groups exhibited VT behaviors that corresponded to their respective stimulus preferences.

The DD group differences in sexual interest align with prior research suggesting that VT can serve as an indicator of sexual interest, particularly in populations with atypical arousal patterns (Mokros et al., 2013). Taken together, these findings provide converging evidence that physiological measures of sexual arousal correspond meaningfully with response latencies interpreted as reflecting sexual interest. By integrating physiological and cognitive processes, this study supports a multi-method approach to assessing sexual deviancy in forensic settings (Marshall & Fernandez, 2000).

Interestingly, these VT differences were significant only for the female stimuli and not the male stimuli. The absence of DD group differences in VT for male stimuli is likely related to the sex-specificity of sexual interest, a well-documented phenomenon wherein sexual interest in a given sex tends to remain consistent across sexual maturity groups (Bailey et al., 2021). Indeed, a recent multidimensional scaling analysis by Bailey et al. (2021) supports this interpretation, such that men’s sexual interest is best represented along two intersecting dimensions: the sex of the target and sexual maturity. Their findings confirm that sexual maturity-based sexual preferences (e.g., attraction to children) are layered onto an underlying sex-based orientation. Accordingly, our finding that group differences emerged only in relation to female stimuli likely reflects our sample composition (i.e., that 69.18% of our sample were heterosexual men who reported acting out sexually primarily against females). Thus, these men’s self-reported sexual preference and viewing-time allocation are consistent with a female-oriented sexual orientation, regardless of maturity level.

### Limitations and Future Directions

Several limitations warrant consideration. First, the potential for social desirability effects cannot be dismissed. As Stinson and Becker (2008) observed, “sexual interests related to deviant, inappropriate, or abnormal stimuli may be minimized or denied in order to avoid stigma, labeling, or perceptions of risk or dangerousness” (p. 380). Although PPG and VT assessments are designed to capture involuntary/indirect processes, participants may still attempt to influence their responses, for example by suppressing arousal or intentionally accelerating or delaying stimulus advancement. This may be especially relevant for civilly committed clients residing in a secure treatment facility who need to demonstrate a reduced risk to sexually re-offend in order to be released. Ciardha et al. (2017) note concerns regarding the potential manipulation of VT tasks, stating that “for those undertaking assessments in adversarial settings, detailed task descriptions are available online; therefore, viewing time alone is not likely to provide a truly reliable measure of sexual interest for forensic assessment” (p. 638).

Both PPG and VT assessments are subject to methodological limitations related to stimulus characteristics and a lack of standardization across studies. Although Affinity images are validated for research purposes, individual images may vary in salience, with some potentially resembling known individuals or differing in perceived attractiveness. Moreover, prior work indicates that VT paradigms differ in stimulus construction, including how categories are defined relative to pubertal stages, degree of sexual explicitness, and whether images are real or computer-generated (Schmidt et al., 2017). Relatedly, the narratives accompanying the PPG images described persuasive and coercive scenarios, whereas the Affinity contains no analogous coercive stimuli. The coercive segments, in particular, may have affected the results, as the sexually violent stories could have elicited greater arousal than the sex and sexual maturity depicted in the corresponding images. Future research should address these limitations by incorporating participant ratings of attractiveness and relevance, developing more tailored stimulus sets, and comparing equivalent stimuli across assessments.

Like the PPG, VT assessments have also been criticized for their lack of standardization regarding scoring algorithms and procedures (Schmidt et al., 2017; Wilson & Miner, 2016). For example, some VT paradigms involve a single exposure to stimuli, whereas others include multiple trials (e.g., initial familiarization followed by a rating trial). Moreover, instruments differ in the types of ratings participants are asked to make. For instance, the Affinity has participants rate the images on a cognitive appraisal (i.e., sexually unattractive to sexually attractive), whereas the Abel Assessment for Sexual Interest (Abel Screening, Inc., 2004) requires an emotional response (i.e., disgusted to aroused), reflecting fundamentally different response processes (Wilson & Miner, 2016). These measures assess physiological sexual arousal (PPG) or inferred sexual interest (VT) and should not be interpreted as indicators of sexual offending behavior.

Sample characteristics and measurement constraints further limit generalizability. The study included only adult males who were civilly committed, restricting conclusions to this population. Replication across lower-risk offenders and community samples is needed to determine whether PPG–VT convergence extends across offender groups and gender identities. Finally, analyses relied solely on mean ranked OTLs as the index of VT. Other indices, such as self-report ratings, raw viewing time, and post-task latency, may capture additional dimensions of sexual interest, and research should work to adopt multi-method VT approaches to provide a more nuanced and robust assessment.

###  Conclusions and Implications

This study provides compelling evidence that DD classifications (based on sexual arousal) produced by the Monarch 21 PPG correspond meaningfully to VT-based measures of sexual interest. Specifically, individuals categorized in the adult arousal group showed longer viewing times for adult stimuli, whereas those in the child arousal group showed longer viewing times for child stimuli. Individuals in the undifferentiated group had longer viewing times for female stimuli compared to male stimuli, suggesting greater salience of sexual orientation rather than age group preference. Thus, our findings suggest that the three-category DD classification captures distinct and psychologically meaningful profiles of sexual interest.

Additionally, our results suggest that cognitive measures such as VT paradigms may serve as valuable adjuncts to more invasive assessments (e.g., PPG), particularly when physiological methods are unavailable or inappropriate. In fact, some studies exploring the discriminative validity of VT assessments have indicated that they can accurately differentiate adult sexual offenders from non-sexual offenders (Gress et al., 2013). Furthermore, a study by Gray and colleagues (2015) found that participants with a higher VRT™ to child stimuli were significantly more likely to be arrested and charged for sexual offenses compared to those with a lower VRT^™^ to child stimuli, with re-offense more likely in the first 6 months. However, more research is needed to explore the predictive utility of VT measures, particularly in relation to sexual recidivism, to determine whether these less invasive tools can independently inform risk assessment and treatment planning.

From a clinical and forensic perspective, our results highlight the value of using the Monarch PPG’s DD classification to inform case conceptualization, treatment, and risk assessment, leading to more targeted interventions. The distinct VT profiles associated with each DD group suggest that these classifications represent more than arbitrary cutoffs; rather, they reflect meaningful differences in how individuals process and evaluate sexual stimuli. Furthermore, the convergence between physiological measures of arousal and cognitively based measures of sexual interest strengthens the case for multimodal assessment in forensic and clinical contexts. In this regard, VT assessments could potentially be used to corroborate PPG findings, particularly when response validity is in doubt or when individuals are unwilling or unable to complete PPG testing.

## Data Availability

The data that support the findings of this study are not openly available due to the presence of potentially identifying or sensitive information embedded.

## Code availability

No custom code was used for the analyses, rather SPSS Version 30 menu options.
